# Discovery of a novel mitochondrial DNA molecule associated with tetrad pollen sterility in potato

**DOI:** 10.1186/s12870-022-03669-8

**Published:** 2022-06-21

**Authors:** Rena Sanetomo, Kotaro Akai, Akito Nashiki

**Affiliations:** 1 Potato Germplasm Enhancement Laboratory, Obihiro University of Agriculture and Veterinary Medicine, Obihiro, Hokkaido 080-8555 Japan; 2 National Agriculture and Food Research Organization, Hokkaido Agricultural Research Center, Memuro, Hokkaido 082-0081 Japan; 3 Graduate School of Science and Technology, The University of Tsukuba, Tsukuba, Ibaraki 305-8572 Japan

**Keywords:** Tetrad sterility, Potato, Cytoplasmic male sterility, Mitochondrial genome, *Solanum stoloniferum*, Nanopore sequencing

## Abstract

**Background:**

Tetrad sterility in potato is caused by a specific cytoplasm, called TSC_sto_, derived from the Mexican wild tetraploid species *Solanum stoloniferum*. Different *S. stoloniferum* accessions crossed as females with *S. tuberosum* resulted in 12 fertile hybrids and 27 sterile hybrids exhibiting tetrad sterility.

**Results:**

Whole-mitochondrial-genome sequencing was performed for two fertile hybrids and three hybrids exhibiting tetrad sterility. Two to seven contigs, with the total assembly lengths ranging from 462,716 to 535,375 bp, were assembled for each hybrid. Unlike for the reference mitochondrial genome (cv. Désirée), two different recombinant-type contigs (RC-I and RC-II) were identified. RC-I featured by the *rpl5-ψrps14* gene joined to the *nad6* gene, generating a novel intergenic region. Using a PCR marker (P-3), we found that this intergenic region occurred exclusively in interspecific hybrids exhibiting tetrad sterility and in their parental *S. stoloniferum* accessions. A part of this intergenic sequence was expressed in the pollen. From a large survey in which P-3 was applied to 129 accessions of 27 mostly Mexican wild species, RC-I was found in diploid *S. verrucosum* and polyploid species. From eight accessions of *S. verrucosum* used as females, 92 interspecific hybrids were generated, in which only those carrying RC-I exhibited tetrad sterility.

**Conclusions:**

RC-I was clearly associated with tetrad sterility, and the RC-I-specific intergenic region likely contains a causal factor of tetrad sterility.

**Supplementary Information:**

The online version contains supplementary material available at 10.1186/s12870-022-03669-8.

## Background

Plant mitochondrial genomes (mitogenomes) are large and complex compared with those of other eukaryotes [1–4]. Mitochondrial genes are mostly conserved and are essential for respiration and metabolism, but mitogenome sizes vary greatly depending on plant species because mitogenomes contain noncoding sequences and sequences of unknown origin [5]. Copy number variation in plant mitochondria results from stoichiometric or heteroplasmic differences [6]. The plant mitogenome sometimes experiences mutations by recombination, insertion, or nuclear invasion in a region favorable for expression. However, the recombinant mitochondrial molecule does not immediately increase in abundance intracellularly. A nuclear gene that controls replication and recombination of the mitogenome exists, and expression of this gene results in increased mitogenome abundance [7]. The interaction between nuclear and mitochondrial genes has an important role in the function and development of reproductive organs. Cytoplasmic male sterility (CMS) is one of the phenomena caused by these interactions [8].

The full structure of potato (*Solanum tuberosum* L.) mitogenomes in the varieties Cicero and Désirée were revealed by Varré et al. [9]. The potato mitogenome has a total length of approximately 474 kb and consists of at least three independent molecules (subgenomes): a 312.5 kb linear molecule and 112.8 kb and 49.2 kb circular molecules. The potato mitogenome is not present as a master circular molecule like it does in other crop species. Instead, different sizes of circular and linear molecules have formed through multiple repeat sequences [9]. Achakkagari et al. [10] compared the mitogenome sequences of 13 accessions of nine cultivated species and one wild species with the previously reported mitogenomes of *S. tuberosum* [9, 11] and *S. commersonii* [12]. Each mitogenome is represented by three independent circular molecules, except in a wild species (*S. bukasovii*), which has a single circular molecule. Some genes are duplicated via repeat sequences in several species, but the number of core genes is similar across all accessions. Another study by Achakkagari et al. [13] revealed that each mitogenome in nine diploid potato clones (mostly *S. tuberosum*) and a wild diploid species (*S. okadae*) consisted of multiple circular molecules with similar structures and gene organization. On the other hand, comparing the mitogenomes of wild and cultivated potatoes, researchers detected DNA polymorphisms in several genes, such as *atp6*, *nad3*, *rps10*, *cob*, and *rpl5-ψrps14* [14], which allowed classification of mitochondrial DNA into α, β and γ types [15]. In addition, stoichiometric variation causing marked copy number differences has been reported for the recombination-derived 10,794-bp sequence harboring the “Band 1” sequence with unknown function existing only in the Mexican wild diploid species *S. verrucossum* and in Mexican polyploid species [16].

Different types of CMS and related genes have been discovered in plants [7, 17–19]. Various CMS types have also been reported in tuber-bearing *Solanum* species [20–23]. Nine cytoplasmic factors in cultivated potato (*S. tuberosum* ssp. *tuberosum*) are involved in sterility traits such as indehiscence, shriveled microspores, no sporadic formation, anther–style fusion, ventral-styled anthers, and thin anthers [24]. The short anther locus (*Sa*), which causes CMS by interacting with *S. phureja* cytoplasm [25], was rediscovered and mapped to chromosome 6 in an inbred line-derived F_2_ population [26]. Seven types of male sterility were detected in interspecific hybrids possessing the *S. verrucosum* cytoplasm, which were likely caused by the interaction of at least four cytoplasmic factors and specific plasmon-sensitive nuclear genes [27].

Tetrad sterile-type cytoplasmic male sterility (T-CMS), in which pollen development is stopped at the tetrad stage and clumps of four premature pollen grains are released, has been observed in interspecific hybrids with cytoplasm derived from the Mexican wild diploid species *S. verrucosum* (2*n* = 2*x* = 24) [25, 27–29] and in cultivars with cytoplasm derived from the Mexican wild tetraploid species *S. stoloniferum* (2*n* = 4*x* = 48) [15, 30, 31]. *S. stoloniferum* is a highly polymorphic wild species distributed from the southern United States of America to Mexico [32]. Among *S. stoloniferum* accessions, only those having a specific cytoplasm TSC_sto_ exhibit T-CMS in interspecific hybrids [33]. TSC_sto_ is associated with an 859-bp mitochondrial DNA fragment amplified from the intergenic region between *rpl5* and *rps10* [33]. Within this intergenic region or in the nearby region, the presence of a part of the *cob* gene (*ψcob*), a pseudogene of the *rps14* gene (*ψrps14*), and a mutation hotspot are known [34, 35]. However, little is known about the causal gene of T-CMS or structural differences in the mitogenome characterizing TSC_sto_.

Previously, we obtained interspecific hybrids from *S. tuberosum* crossed with different *S. stoloniferum* accessions as females, resulting in either fertile hybrids or tetrad sterile-type cytoplasmic male-sterile hybrids (hereafter referred to as T-CMS hybrids or clones) [33]. In this study, the mitogenomes of two fertile hybrids and three T-CMS hybrids were sequenced using Nanopore sequencing technology. Unlike in the reference mitogenome (cv. Désirée), we identified a recombinant-type mitochondrial DNA molecule RC-I specifically found in TSC_sto_. RC-I had a novel intergenic region between *rpl5-ψrps14* and *nad6*. The same region was found in the Mexican diploid *S. verrucosum* and polyploid species. Consequently, a survey of 92 diploid interspecific hybrids generated from eight accessions of *S. verrucosum* as females indicated that only those carrying this intergenic region exhibited T-CMS, demonstrating that the RC-I-specific intergenic region is associated with T-CMS.

## Results

### Constructed contigs and their homology to the reference mitogenome

Nanopore sequencing using MinION was performed for the mitogenomes from two pollen-fertile clones (17H117-9 and 17H131) and three T-CMS clones (15H156, 18H255, and cv. Alwara). All these clones have *S. stoloniferum*-derived cytoplasms: 17H131 has D/α-type cytoplasm, 15H156 and 18H225 have D/γ-type cytoplasm, and 17H117-9 and cv. Alwara have W/γ-type cytoplasm [33]. After base calling and elimination of low-quality reads, the mean quality score (QS) was approximately 11.3 for all the samples, and the read N50 ranged from 14.8 kb to 43.6 kb (Table S1). Out of 28,786 to 1,896,605 high-quality reads, 4071 to 9899 randomly chosen reads mapped to the reference mitogenome of cv. Désirée, which consisted of a 312.5 kb linear molecule (MN104801, hereafter referred to as M01) and 112.8 kb (MN104802, M02) and 49.2 kb (MN104803, M03) circular molecules [9], were assembled into two to seven contigs for each clone (Table 1). The total assembly lengths ranged from 462,716 bp for cv. Alwara to 535,375 bp for 17H131, covering 97.5% (cv. Alwara) to 112.8% (17H131) of the reference mitogenome (Table S1).

**Table 1:** Constructed contigs and a comparison of them and the reference mitogenome of cv. Désirée

Clone	Contig	Length (bp)	No. of genes^1)^	Corresponding region present in
Pollen-fertile clone				
17H117-9	Contig 1	49,826	6	M01
	Contig 2	63,042	7	M01
	Contig 3	45,101	7	M01
	Contig 4	17,176	7	M02
	Contig 5	66,955	11	M02
	Contig 6	187,226	23 (11)	M01 + M02
	Contig 7	49,115	7	M03
	Total	478,441		
17H131	Contig 1	49,807	6	M01
	Contig 2	37,273	10	M01
	Contig 3	245,122	31 (3)	M01
	Contig 4	154,071	22 (10)	M02
	Contig 5	49,102	7	M03
	Total	535,375		
T-CMS clone				
15H156	Contig 1	125,611	18 (1)	M01
	Contig 2	307,654	39 (5)	M01 + M02
	Contig 3	98,254	7 (7)	M03
	Total	531,519		
18H225	Contig 1	264,271	31 (1)	M01
	Contig 2	193,129	34	M01 + M02
	Contig 3	48,791	6	M03
	Total	506,191		
Alwara	Contig 1	413,590	48 (5)	M01 + M02
	Contig 2	49,126	7	M03
	Total	462,716		

^1)^ Open-reading frames (*orfs*) and tRNA genes were not counted. Duplicated genes are shown in parentheses.

**Fig. 1:**
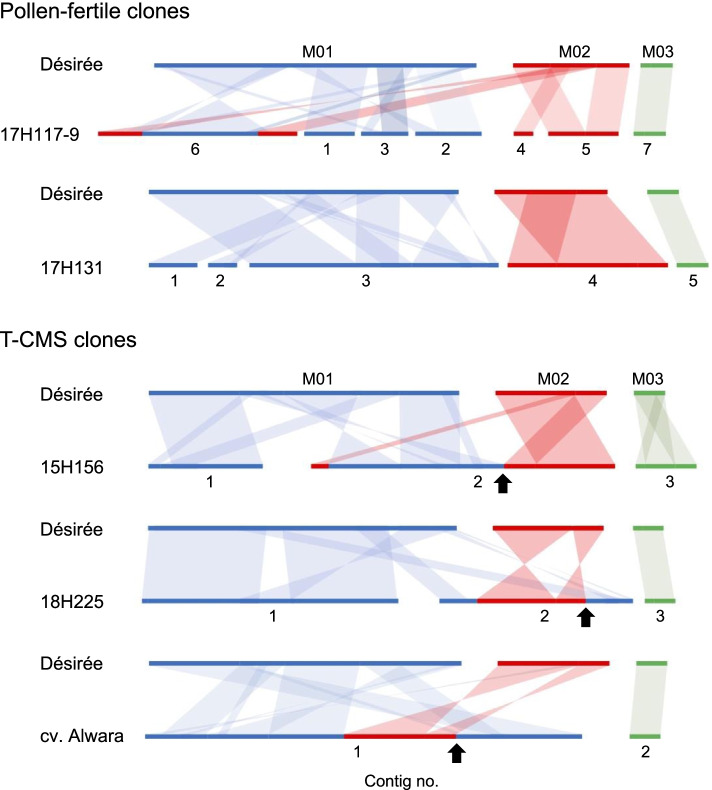
Schematic comparisons of assembled contigs, with Désirée showing an abundance of genomic rearrangements. The blue, red, and green regions are homologous to the M01, M02, and M03 reference mitogenome regions, respectively, of cv. Désirée. The arrows indicate RC-I-specific intergenic regions

Sequence homology with the reference mitogenome sequences was investigated by dot-plot analysis using D-GENIES with the default parameters (Fig. S1), and the results are shown in Fig. 1. The five mitogenomes were structurally different from the reference mitogenome and from each other. Complex rearrangements with frequent inversions and translocations and some duplications were visualized. M03 of cv. Désirée was maintained as a single intact molecule (shown in green in Fig. 1) in all clones except 15H156.

### Gene annotation

The gene annotation results are presented in Additional file 3. The total numbers of protein-coding genes, their truncated ones, and rRNA genes varied among the clones (Table 1). Of these genes, 36 standard mitochondrial genes (*atp1*, *atp4*, *atp6*, *atp8*, *atp9*, *ccmB*, *ccmC*, *ccmFC*, *ccmFN*, *cob*, *cox1*, *cox2*, *cox3*, *matR*, *mttb*, *nad1*, *nad3*, *nad4*, *nad4L*, *nad5*, *nad6*, *nad7*, *nad9*, *rpl2*, *rpl5*, *rpl10*, *rpl16*, *rps1*, *rps3*, *rps4*, *rps10*, *rps12*, *rps13*, *rps19*, *sdh3*, and *sdh4*) and 5S, 18S, and 26S rRNA genes (*rrn5*, *rrn18*, and *rrn26*) were identified in all five clones except for 15H156, which lacked *rpl16* (see Additional file 3). However, *nad2*, which is common in the potato mitogenome [9, 10], was not found in every clone. In addition to the genes with complete sequences, duplicated or truncated and fragmented sequences of these genes were also identified. Four truncated genes, namely, *atp8* (72 bp), *cox2* (104 bp), *nad6* (66 bp), and *rps1* (90 bp), were found in all except 17H117-9, which lacked all these truncated genes.

M03 of cv. Désirée and equivalent molecules, shown in green in Fig. 1, consisted of a set of seven genes (*rpl2*, *rpl10*, *atp9*, *ccmB*, *nad9*, *ccmFN*, and *sdh3*), except in the case of 18H225, which lacked *atp9* from this gene set. 15H156 had a double-sized contig (contig 3), in which the set of seven genes was duplicated.

**Fig. 2:**
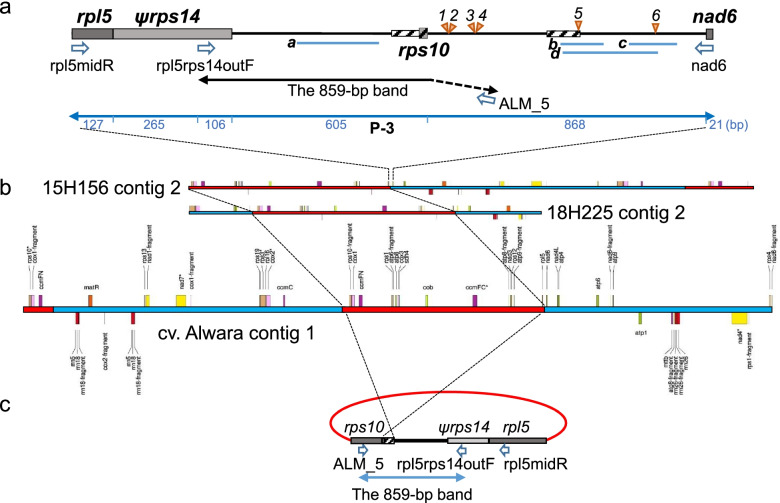
Schematic representation of the recombinant contig RC-I. **a** The P-3 region in cv. Alwara, amplified using the primer pair rpl5midR/nad6 (1992 bp). The positions of nucleotide substitutions and insertions/deletions described in Table 2 are shown in *Italic* figures. Homologous regions with four reads (*a* to *d*) from pollen transcripts are shown by blue lines. A108-bp region and a 105-bp region with high homology (> 94%) to those in M02 are shown by striped boxes. The 108-bp sequence contained the first 25 bp sequence of *rps10*. **b** RC-I in three T-CMS clones. **c** Hypothesized circular molecule that amplified the 859-bp band and coexisted substoichiometrically with RC-I

The other mitochondrial genes were located in one (cv. Alwara) to six contigs (17H117-9) in each clone, with a total of 15 different contigs. Seven of the 15 contigs were terminated with either *rpl5-ψrps14*, *nad6* or *rps10* (see Additional file 3). *ψrps14*, located downstream of *rpl5*, is a pseudogene previously described both in potato and in Arabidopsis [9, 14, 35–37], and it has been shown that a functional copy of the gene has been transferred to the nucleus [38] and that the sequence of the mitochondrial pseudogene has remained virtually unchanged for the past 80 million years [39]. Genes identified in contigs 1, 2 and 3 of 17H117-9; contigs 1, 2 and 3 of 17H131; contig 1 of 15H156, and contig 1 of 18H225 were all found in M01 of cv. Désirée. These contigs are represented in blue in Fig. 1. Similarly, genes identified in contigs 4 and 5 of 17H117-9 and contig 4 of 17H131 were all found in M02 of cv. Désirée. These contigs are represented in red in Fig. 1. Interestingly, recombinant-type contigs that included the genes found in M01 and M02 were identified (Table 1; Fig. 1). These recombinant contigs (RCs) were classified into two types (RC-I and RC-II). RC-I was found in contig 2 of 15H156, contig 2 of 18H225, and contig 1 of cv. Alwara (all of which are T-CMS clones), which contained a 108.3 kb highly conserved sequence consisting of 15 protein-coding genes (*rps10*, *cox1*, *ccmFN*, *sdh3*, *rps1*, *atp6*, *atp8*, *cox3*, *sdh4*, *cob*, *ccmFC*, *nad3*, *rps12*, *atp9*, and *rpl5-ψrps14*). This conserved sequence contained a set of genes similar to a set within M02 of cv. Désirée but differed by the two inversions (Fig. 1). The *rpl5-ψrps14-*end of this sequence (shown by arrows in Fig. 1) was always joined to the last 468 bp of *nad6*, a member gene of M01 (Fig. 2a). The full length *nad6* (654 bp) was present in the fertile clones and one T-CMS clone (18H225), while the truncated 468 bp *nad6* was found only in T-CMS clones and joined to *rpl5-ψrps14* (see Additional file 3). The other end of this conserved sequence was terminated at 15H156 or joined to different member genes of M01 in 18H225 and cv. Alwara (Fig. 2b). The second recombinant type (RC-II) was found in contig 6 of 17H117-9 (pollen fertile), in which the conserved sequence found in RC-I was fragmented into different contigs, and the *rpl5-ψrps14* end was not joined to *nad6* (Additional file 3).

### Characterization of the *rpl5*-*ψrps14*–*nad6* connecting region specific to RC-I

Since we previously found a diagnostic PCR marker for TSC_sto_ in a location near *rpl5* [33], the RC-I-specific intergenic region between *rpl5-ψrps14* and *nad6* in cv. Alwara was specifically investigated. According to the results of a nucleotide homology search using BLASTN (NCBI), there were 108-bp and 105-bp noncoding regions highly homologous (> 94%) to those in M02 (Fig. 2a, striped boxes). The 108-bp sequence ended with the first 25-bp sequence of *rps10*. There is a truncated copy of *cob* (*ψcob*) between *rpl5-ψrps14* and *rps10* in cv. Désirée [9] and in nine diploid breeding clones except *S. okadae* [13]. Like in *S. okadae*, there was no *cob*-like sequence in this region in our materials. Interestingly, the 105-bp noncoding region was 83% homologous to a 541-bp region of the mitogenome of the *Capsicum annuum* cultivar CMS line FS4401 (Sequence ID: KJ865409.1), the region of which encodes *orf165 *[40].

To further investigate the RC-I-specific region, four PCR primer sets were designed (P-1 to P-4, shown in Table S2). P-1 and P-2 amplify the intragenic regions of *rpl5* and *nad6*, respectively, while P-3, using the primer set rpl5midR/nad6, amplifies the RC-I-specific region consisting of the last 127 bp of *rpl5*, *ψrps14*, the intergenic region, and the first 21 bp of the truncated *nad6* gene (Fig. 2a). P-4 amplifies the intergenic region between *rps4* and *nad6*. *rps4* was always joined to *nad6* according to the gene annotation data (Additional file 3), and both genes are in the same transcription unit [9]. Thirty-eight accessions of *S. stoloniferum* and 39 interspecific hybrids resulting from *S. stoloniferum* × *S. tuberosum* were analyzed via these primer sets. The gene-specific primer sets P-1 and P-2 and the P-4 primer set amplified single bands with expected sequence lengths (534, 468, and 919 bp, respectively) from all the samples (Table S3). However, P-3 amplified approximately 1.9 kb bands from 18 accessions of *S. stoloniferum* and 27 interspecific hybrids derived from 12 accessions of *S. stoloniferum*. All these hybrids exhibited either T-CMS or the other type of pollen sterility [33]. The remaining 20 accessions of *S. stoloniferum* and 12 hybrids derived from six accessions of *S. stoloniferum* showed no amplified band when P-3 was used; these 12 hybrids produced normal pollen [33]. Therefore, the region amplified by P-3 was specifically associated with the T-CMS trait (Table S3).

The P-3-amplified bands from 17 accessions of *S. stoloniferum*, 27 interspecific hybrids, and cv. Alwara were sequenced. The total length ranged from 1992 to 2010 bp due to the various lengths of intergenic regions, which ranged from 1844 to 1862 bp. Four nucleotide substitutions, one 3-bp insertion/deletion, and one 15-bp duplication were detected (Table 2). Based on these polymorphisms, five different sequences were identified. Cv. Alwara had the shortest sequence (1992 bp; see Fig. S2), which was the most frequent sequence in *S. stoloniferum* (8 of 17 accessions).

**Table 2:** Polymorphisms in the region amplified by P-3 (*rpl5*-*ψrps14*–*nad6*) in *S. stoloniferum* and interspecific hybrids with TSC_sto_ cytoplasm

Species or hybrid family^1)^		Polymorphism and its position in parentheses^2)^						Size (bp)
	Accession or pedigree	1 (1165/1166)^3)^	2 (1166)	3 (1247)	4 (1252)	5 (1575/1576)^3)^	6 (1807)	
*S. stoloniferum*	PI 255547, PI 498030, PI 498038, PI 558395, PI 558449	CTT	A	A	A	TCTTCCACCTCGACT	A	2010
15H156 (1)	F_1_ (PI 255547 × 10H17)							
19H81 (1)	F_1_ (PI 498038 × 10H17)							
19H69 (1)	F_1_ (PI 558449 × Konafubuki)							
*S. stoloniferum*	PI 497994, PI 498035	ATT	C	C	T	TCTTCCACCTCGACT	A	2010
19H71 (2)	F_1_ (PI 497994 × 10H17)							
19H72 (3)	F_1_ (PI 497994 × 10H17)							
19H73 (3)	F_1_ (PI 497994 × 10H17)							
19H74 (3)	F_1_ (PI 497994 × 10H17)							
19H75 (2)	F_1_ (PI 498035 × 10H17)							
19H76 (3)	F_1_ (PI 498035 × 10H17)							
19H77 (1)	F_1_ (PI 498035 × Konafubuki)							
*S. stoloniferum*	PI 545726	TTT	A	A	A	-	A	1995
*S. stoloniferum*	PI 558455	-	A	C	T	-	A	1992
*S. stoloniferum*	PI 161178, PI 275248, PI 310964, PI 545780, PI 545788, PI 558447, PI 558450, PI 558451	-	A	C	T	-	G	1992
17H120 (6)	F_1_ (PI 558450 × 10H17)							
17H121 (1)	F_1_ (PI 558450 × Konafubuki)							
Alwara	4*x* cultivar							

^1)^ Number of hybrids within the family

^2)^ Polymorphic sites are shown in Fig. 2a. Their positions in cv. Alwara are indicated in terms of the number of base pairs from the 5’-end of the rpl5midR primer

^3)^Insertion position

### Homology between the P-3-amplified region and the 859-bp band

Previously, we identified the 859-bp band, which was amplified using the primer pair rp15rps14outF/ALM_5; this band ultimately served as a diagnostic marker for TSC_sto_ [33]. The sequence of the first 711 bp of the 859-bp band completely matched the region from 393 to 1103 bp of the P-3-amplified sequence and was located within the intergenic region between *rpl5* and *nad6* (Fig. 2a). The last 25 bp of the common 711-bp sequence was identical to the first 25 bp of *rps10*. The remaining 148 bp of the 859-bp band was not homologous to any part of the intergenic region, whereas it exhibited 100% homology with *rps10* of cv. Désirée. This finding can be explained by the primer location of ALM_5, which was designed by Lössl et al. [15] to bind to *rps10*. Therefore, the 859-bp band and the P-3 sequence shared a 686-bp intergenic sequence plus the first 25-bp sequence of *rps10* in common; then, the 859-bp band continued the sequence of *rps10*, while the P-3 sequence was joined to the 468-bp truncated *nad6* sequence with a spacer region (868 to 886 bp in size) (Fig. 2a).

**Fig. 3:**
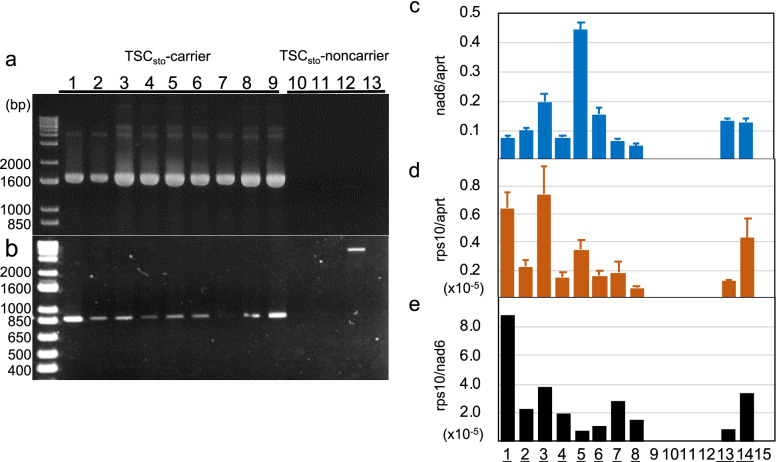
Stoichiometric differences between P-3 and the 859-bp band. Electrophoresed gels of the PCR-amplified bands via (**a**) rpl5rps14outF/nad6 and (**b**) rpl5rps14outF/ALM_5 (for the 859-bp-band) primer sets. 1, cv. Alwara; 2, 18H225; 3, 15H156; 4, 19H69; 5, 19H77; 6, 19H72-1; 7–9, *S. stoloniferum* PI 498030, PI 545726 and PI 558455, respectively; 10, 17H117-9; 11, 17H131; 12, 10H6-1; 13, 17H125-1. A 1 kb Plus DNA ladder (Invitrogen™) was used as a size marker in the first lane. Full-length gels are presented in Supplementary Fig. S4. **c–e** Amplification differences detected via real-time PCR. Relative quantities compared with those of aprt (adenine phosphoribosyl transferase, a housekeeping nuclear gene; Nicot et al. [41]) were measured for rpl5-nad6 (**c**) and rpl5-rps10 (**d**), and their differences are shown as the relative quantities of rpl5-rps10 divided by those of rpl5-nad6 (**e**). 1–5, *S. stoloniferum* PI 497994, PI 498038, PI 545780, PI 558450 and PI 558455, respectively; 6, 15H156; 7, 17H120-1; 8, 18H225; 9, 17H118-3; 10, 17H117-9; 11, 17H131; 12 and 13, *S. verrucosum* PI 275260 and PI 498061, respectively; 14, cv. Alwara; 15, cv. Désirée. The samples with TSC_sto_ are underlined

PCR amplification between *nad6* and *rps10* via the primer pair nad6/ALM_5 or several other newly designed primer pairs failed, indicating that *nad6* is not joined to *rps10*, with the exception of the 25-bp *rps10* remnant. Using the primer pairs rp15rps14outF/nad6 (internal P-3) and rp15rps14outF/ALM_5 (for the 859-bp band), we amplified sequences from TSC_sto_-carrying *S. stoloniferum* and its interspecific hybrids and TSC_sto_-noncarrying interspecific hybrids (Fig. 3). The primer pair rp15rps14outF/nad6 yielded strong bands exclusively from TSC_sto_-carrying plants, while the primer pair rp15rps14outF/ALM_5 yielded faint bands with 859 bp in size from TSC_sto_-carrying plants and a > 2 kb band from one pollen-fertile clone. These results indicated that the two primer sets amplified different molecules with different copy numbers. To confirm the copy number differences, real-time PCR was performed in conjunction with the same forward primer but with two different reverse primers: one designed to target *rps10* (rpl5-rps10 primer set) and the other designed to target the spacer region toward *nad6* (rpl5-nad6 primer set). Relative quantities compared with those of the adenine phosphoribosyl transferase gene (*aprt*), a housekeeping gene encoded in the nuclear genome [41], were measured. The relative quantities when rpl5-nad6 was used varied from 0.05 to 0.44 (Fig. 3c), while those when rpl5-rps10 was used varied from 0.073 × 10^–5^ to 0.74 × 10^–5^ from TSC_sto_-carrying plants (Fig. 3d). Thus, rpl5-rps10 amplified much less mitochondrial DNA than rpl5-nad6 did (1 vs. 0.8 × 10^5^–8.8 × 10^5^, Fig. 3e). Mitochondrial DNA of the pollen-fertile clones (TSC_sto_-noncarriers) was not amplified at all when these two primer sets were used.

### Expression analysis of the intergenic region between *rpl5*-*ψrps14* and *nad6*

To search for novel candidate genes associated with T-CMS, a pollen transcriptome analysis was performed on the RC-I-specific intergenic region between *rpl5-ψrps14* and *nad6*. Total pollen RNA from two *S. stoloniferum* accessions, both with TSC_sto_, was extracted, and a 150 bp paired-end reads were generated via Illumina sequencing. A total of 17,624 reads from *S. stoloniferum* PI 498035 (2010 bp in size for P-3) and a total of 12,593 reads from *S. stoloniferum* PI 558450 (1992 bp in size for P-3) were mapped to the 413,590-bp contig 1 of cv. Alwara (Table S4). Two and six reads of PI 498035 were mapped to *rpl5* and *nad6*, respectively, while no reads of PI 558450 were mapped to either gene. Three reads from PI 498035 (*a*, *b*, and *c* in Fig. 2a) and one read from PI 558450 (*d*) were mapped to the intergenic region between *rpl5-ψrps14* and *nad6* (Table S4). Reads *b*, *c* and *d* overlapped with each other and occupied a 363-bp sequence in the last part of the intergenic region. One nucleotide substitution (A/G at 1807; Table 2) and a 15-bp duplication (between 1575 and 1576; Table 2) were confirmed in the mapped reads. Read *a* (255 bp) was mapped to the homologous region between the intergenic region and the 859-bp band.

### Polymorphisms in the *cox2*-*rps1* connecting region specific to RC-II

**Fig. 4:**
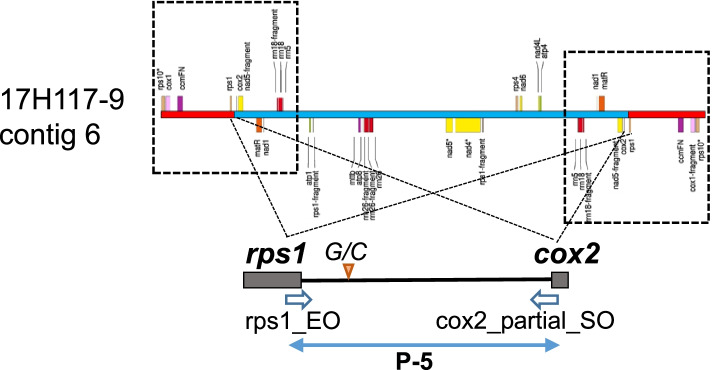
Schematic representation of the P-4 region in contig 6 of 17H117-9, amplified using the primer pair rps1_EO/cox2_partial_SO (1715 bp). The dashed frames indicate symmetrically duplicated regions (46.6 kb)

The second recombinant type RC-II was found in contig 6 of 17H117-9 (pollen fertile). The central region of contig 6 consisted of genes of M01. However, both terminal sequences (each 46.6 kb in size) were symmetrically arranged with genes of M01 and M02. The connecting region consisted of *rps1* of M02 and the *cox2-*partial gene of M01. A PCR primer set (P-5) (Table S2) was designed to amplify the RC-II-specific region consisting of the last 98 bp of *rps1*, the intergenic region, and the first 24 bp of *cox2* (Fig. 4).

The same set of 38 accessions of *S. stoloniferum* and 39 interspecific hybrids were analyzed using P-5. Single 1715-bp bands were amplified from 18 accessions of *S. stoloniferum*, of which five accessions also had the P-3 band. The 1715-bp band was amplified from six interspecific hybrids derived from three accessions of *S. stoloniferum*. These hybrids did not exhibit T-CMS [33], indicating that the 1715-bp band was not associated with the T-CMS trait or with the cytoplasmic type (Table S3). The 1715-bp band amplified from the 24 samples was sequenced, the results of which revealed only one nucleotide substitution from G to C at a position 333 bp from the top of the rps1_EO primer in only one accession of *S. stoloniferum* (PI 498000) and its hybrid (Tables S3, S5; Fig. S3).

### Distribution of RC-I and RC-II in tuber-bearing *Solanum* species

The presence or absence of recombinant mitogenomes (RC-I and RC-II) was surveyed by the use of the P-3 and P-5 primer sets for 129 accessions of 27 mostly Mexican wild species in addition to the 38 accessions of *S. stoloniferum* described above (Table S5).

The P-3 band was detected for *S. verrucosum* (3 of 10 accessions, Verrucosa Group), *S. hjertingii* (6 of 7 accessions, Longipedicellata Group) and five species of the Iopetala/Acaulia Group, namely, *S. guerreroense* (1 of 2 accessions), *S. hougasii* (7 of 7 accessions), *S. iopetalum* (1 of 15 accessions), *S. schenckii* (4 of 7 accessions), and *S. demissum* (1 of 23 accessions). With respect to the remaining 106 accessions, clear P-3 bands were not detected, although faint bands with the same size were sometimes detected, albeit inconsistently.

The P-5 band was detected for *S. bulbocastanum* (2 of 2 accessions, Bulbocastana Group), *S. hjertingii* (2 of 7 accessions, Longipedicellata Group), and *S. iopetalum* (4 of 15 accessions, Iopetala Group). Only one accession of *S. hjertingii* (PI 186559) yielded both P-3 and P-5 bands (Table S5).

### Association of RC-I with T-CMS in *S. verrucosum*

Since RC-I, which was associated with T-CMS in *S. stoloniferum*, was discovered in the diploid species *S. verrucosum*, plants from eight accessions of *S. verrucosum* were pollinated with pollen from two or three diploid clones (mostly *S. phureja*). Of these accessions, only PI 498061 had RC-I according to the P-3 marker assay results. A total of 92 interspecific hybrids, with nine to 15 hybrids per accession, were examined for pollen shape and stainability. All but ten hybrids exhibited high pollen stainabilities, with family means ranging from 59.0% to 79.0% (Table 3). The ten hybrids were all derived from PI 498061 and showed T-CMS. Hence, the association of RC-I with a T-CMS trait was verified in the *S. verrucosum* hybrids. The copy number difference between molecules generating the P-3 band and the 859-bp band was also confirmed via real-time PCR, in which a 90,000-fold difference was found for *S. verrucosum* PI 498061 (Fig. 3e).

**Table 3:** Stainability of pollen from interspecific hybrids resulting from crosses between *S. verrucosum* (female) and 2*x *clones (male)

Female accession	No. of male parents	No. of hybrids	Pollen stainability (%)		Pollen shape
			Average	SD	
PI 275260	3	14	73.3	17.60	Normal
PI 498061	3	10	2.8^1)^	2.51	T-CMS
PI 545745	2	10	79.0	19.30	Normal
PI 545746	2	9	73.7	15.83	Normal
PI 545747	2	9	67.2	13.90	Normal
PI 545810	3	15	67.0	7.63	Normal
PI 545811	2	10	62.7	22.78	Normal
PI 558488	3	15	59.0	14.57	Normal

^1)^ One of four pollen grains in tetrad pollen was stained

## Discussion

### Mitogenome assembly

In this study, five mitogenomes were assembled via 4000 to 10,000 Nanopore long reads, resulting in two to seven contigs for each clone, with total lengths ranging from 463 to 535 kb. Recently, three individual molecules in each of 12 potato accessions from diverse taxa were reported by Achakkagari et al. [10]. Similarly, Varré et al. [9] reported three autonomous molecules, namely, two circular (M02 and M03) and one linear (M01) DNA molecules, for two *S. tuberosum* cultivars, and Cho et al. [11] reported five circular DNA molecules in *S. tuberosum*, in which three of them had shared sequences. In the present study, only M03 was retained as a single molecule in all the clones, whereas M01 and M02 were rearranged intensively by inversions, translocations, and duplications and formed one to six contigs in each clone. Structural variability via repeat sequences is remarkably large in plant mitogenomes [8, 13]. The larger number of contigs was constructed for 17H117-9 and 17H131, probably because branching molecules often caused sequence termination at the ends of these contigs [9, 13].

### T-CMS is associated with the intergenic region between *rpl5*-*ψrps14* and *rps10*

We discovered a recombinant-type mitochondrial molecule (RC-I) in T-CMS hybrids of *S. stoloniferum* carrying TSC_sto_ and their parental accessions. In this recombinant-type molecule, a novel intergenic sequence between *rpl5-ψrps14* and *nad6* was found. *rpl5-ψrps14* and *nad6* are often located in different molecules [9, 13]. Seven contigs assembled in the present study were terminated with *rpl5-ψrps14*, *rps10*, or *nad6*. This implies that the noncoding spacer regions around these genes were often branched or rearranged and coexisted, resulting in the assembly of multiple smaller contigs or a large linear contig. Quiñones et al. [37] reported alternative rearrangements downstream of *rpl5-ψrps14,* in which *rps10* is present in species other than *S. tuberosum*, whereas *ψcob* is present only in *S. tuberosum*. The former and latter types correspond to α- and β-type mitochondrial genomes, respectively, in accordance with the classification of Lössl et al. [34]. Translocation of *ψcob* downstream of *rpl5-ψrps14* was also found in the mitogenomes of *S. stenotomum*, *S. phureja*, and *S. bukasovii* [13] and was found to be rather frequent in tuber-bearing *Solanum* species [14]. An approximately 1.5-kb R4 repeat overlaps the *ψcob* sequence [9, 13]. Thus, the region between *rpl5-ψrps14* and *rps10* has been known as a recombination hot spot in *Solanum* species via the R4 repeat sequence [13–15, 34, 35, 42–44]. Therefore, the mitogenome of TSC_sto_ is obviously one of the recombination variants detected via this recombination hot spot.

### The 859-bp band derived substoichiometrically from a molecule that underwent fission

The 859-bp band was identified as a diagnostic marker for TSC_sto_ by Sanetomo and Nashiki [33]. The 859-bp band showed complete homology in the first 711-bp sequence with the RC-I-specific region between *rpl5* and *nad6* (the P-3 region). However, we revealed that the P-3 region and the 859-bp band were amplified from different molecules with different copy numbers. The 108.3-kb region homologous to the M02 region in the recombinant contig (RC-I) was highly conserved among T-CMS clones and terminated with *rpl5-ψrps14* on one end and with *rps10* on the other end. Therefore, we suggest that the 859-bp band was amplified from a molecule that resulted from fission, derived by the joining of both ends of the conserved region, resulting in a circular molecule (Fig. 2c). Because of the low copy number, circular structure, and complete homology between the circular molecule and the M02 region in RC-I, the assembly algorithm used in this study was probably unable to detect this circular sequence as an independent contig.

Although many DNA rearrangements and polymorphisms in mitochondrial DNA have been detected via PCR in somatic hybrids [34, 35, 43], these two molecules, namely, RC-I and its internal sequence-derived circular molecule, have not been discovered. The two molecules coexisted in cells with TSC_sto_, but the circular molecule was 100,000 times rarer in the cells (Fig. 3e). Copy number variation, substoichiometric variation, and fusion- and fission-type molecules occurring by recombination are frequent in plant mitogenomes [45–48]. Thus, compared with the reference mitogenome of cv. Désirée, RC-1 is likely a fusion-type molecule (relating to tetrad sterility), and the circular molecule is a fission-type molecule, both of which are present in a heteroplasmic state.

### Candidate gene for T-CMS

New open reading frames (*orfs*) generated by recombination in the mitogenome are often associated with CMS. Many of them have a chimeric structure with genes encoded in the mitogenome [7, 49, 50]. Achakkagari et al. [13] found *orf210* in the intergenic region between *rpl5-ψrps14* and *rps10* in a cultivated potato carrying W/α cytoplasm. In the intergenic region between *rpl5-ψrps14* and *nad6* in RC-I, a 105-bp sequence showed homology with *orf165* of a CMS hot pepper line [40]. We identified four reads of pollen transcripts derived from this region, suggesting the presence of a novel *orf*. However, the number of reads mapped to this region was too small to accurately localize the *orf*. This occurred mainly because interspecific hybrids did not provide sufficient pollen grain due to T-CMS, and thus, mature, normal pollen from *S. stoloniferum* was used in this study.

The *CMS-WA* gene in rice, located in the same intergenic region found in this study, is expressed specifically at the pollen mother cell (PMC) stage and induces CMS by interacting with *Cox11* to cause premature death of tapetum cells [51, 52]. The Arabidopsis *quartert* (*qrt*) mutant releases premature pollen in the form of tetrads [53–55], which is similar to the T-CMS phenotype in our study. In this mutant, proper degradation of the cell wall of the PMC after meiosis and subsequent segregation of microspores fail [53, 56]. Shishova et al. [57] detected metabolic changes in anthers in T-CMS potato varieties compared with normal pollen-producing varieties. Therefore, it can be assumed that the transcripts of the causal gene for CMS accumulate in the tapetal tissues and PMCs and suppress pollen development. Reduced accumulation of the transcripts allows the production of normal pollen in wild species and fertility-restored lines even if these plants have a gene causing CMS [58–63]. These findings are in accordance with our results in that only a few transcripts were mapped in normal pollen tissue of *S. stoloniferum*. To identify the causal gene, it is necessary to conduct a transcriptome analysis during early development (encompassing PMCs to tetrad pollen) in the T-CMS clones.

### Origin of TSC_sto_

Using P-3, we surveyed a large panel of accessions (mostly of Mexican wild species) for the presence of the recombinant-type molecule RC-I. RC-I was frequent among Mexican tetraploid and hexaploid species and was found in three of ten accessions of the Mexican diploid species *S. verrucosum.* The shared nature of cytoplasmic factors among these species has also been reported for chloroplast DNA polymorphisms [64, 65], a mitochondrial DNA-derived amplified band “Band 1” [16], and the 859-bp band [33]. Scotti et al. [14] classified mitogenomes in 53 genotypes of 30 cultivated and wild potato species into 13 haplotypes of 7 subgroups according to mutations in the region containing *rps10*, *cob*, and *rpl5-ψrps14* and classified *S. verrucosum*, *S. stoloniferum* and *S. demissum* into the same group. These findings support the idea that *S. verrucosum* is a cytoplasmic donor for Mexican polyploid species [16, 33, 64, 65]. Therefore, it was suggested that TSC_sto_ originated from *S. verrucosum* [33] because the cytoplasm of *S. verrucosum* also causes T-CMS [25, 27, 29].

We obtained interspecific hybrids from both P-3 target-carrying and P-3 target-noncarrying accessions of *S. verrucosum* as female parents crossed with diploid potato plants. The T-CMS phenotype occurred only for the hybrids generated from the P-3 target-carrying *S. verrucosum* accession, which strongly supports that the intergenic region of RC-I is responsible for T-CMS and that TSC_sto_ originated from *S. verrucosum*.

### RC-II derived from *S. bulbocastanum*?

*S. stoloniferum* is an allotetraploid species with the genome constitution of AABB [66–70]. *S. verrucosum* is the A genome maternal progenitor of *S. stoloniferum* [16, 64, 65, 70]. The B genome might be homologous to the genomes of Mexican diploid species *S. cardiophyllum*, *S. ehrenbergii*, *S. jamesii*, or *S. bulbocastanum* [67, 68, 70, 71]. The P-5 band specific to RC-II was observed only in *S. bulbocastanum* among the diploid species evaluated, implying that, as a maternal progenitor to *S. stoloniferum*, *S. bulbocastanum* contributed to the B genome, as suggested by the shared harboring of late blight resistance genes [71, 72] and root-knot nematode resistance genes [73]. Since *S. stoloniferum* is a highly polymorphic species [32], different maternal progenitors (*S. verrucosum* or *S. bulbocastanum*) might provide different nuclear genomes (A or B) and form a superspecies of sorts, such as *S. stoloniferum*. In fact, not all *S. stoloniferum* accessions have cytoplasm that causes T-CMS [33], and the same was true for *S. verrucosum* (Table 3). However, chloroplast DNA analyses revealed clear differences between *S. stoloniferum* and *S. bulbocastanum* [64, 65]. In the present study, one *S. hjertingii* and six *S. stoloniferum* accessions yielded both P-3 and P-5 bands, clearly indicating that the PCR bands alone cannot be used to deduce the cytoplasmic origin of those accessions. Whole-genome sequences and structural variations of mitogenomes in *S. bulbocastanum* are needed to resolve this.

## Conclusion

We discovered that the recombinant mitogenome RC-I was associated with T-CMS in interspecific hybrids of *S. stoloniferum* and that the intergenic region between *rpl5-ψrps14* and *nad6* was most likely a causal region for T-CMS. We also found the same mitogenome RC-I in a limited number of accessions of *S. verrucosum*, which resulted in its hybrids exhibiting T-CMS. Since *S. stoloniferum* has been widely used as a genetic resource for disease and pest resistance in potato breeding [74, 75], TSC_sto_ has already been introduced into potato varieties worldwide [15, 76–78], and materials with TSC_sto_ are increasing and gradually replacing pollen-producing parents in cross-breeding [76, 77, 79]. The P-3 marker facilitates the identification of TSC_sto_ more accurately than does the previously reported marker (the 859-bp band [33]), which is helpful for control cross-breeding.

However, T-CMS may be a useful trait for efficient seed production in diploid inbred-based hybrid breeding, providing that the functions of T-CMS and a pollen-fertility restoration mechanism are elucidated [23]. A dominant male fertility restorer (*Rt*) gene, located very distal from the centromere, has been found for CMS caused by *S. tuberosum* cytoplasm [31, 80]. In our recent study (unpublished data), some of the interspecific hybrids resulting from *S. stoloniferum* × *S. tuberosum* backcrossed with the *S. stoloniferum* parent show pollen stainability with acetocarmine, indicating the presence of a pollen fertility restoration mechanism. However, the fertility and morphology of the pollen were highly influenced by environmental conditions, and polyploidy and aneuploidy hinder the identification of the restoring factor(s). Alternatively, T-CMS caused by RC-I in the diploid species *S. verrucosum* could facilitate the exploration of the mechanism driving CMS and the discovery of a pollen fertility restoration gene.

## Methods

### Plant materials and DNA samples

The plants used for whole-genome sequencing included three F_1_ hybrid plants, namely, 15H156 (*S. stoloniferum* PI 255547 × 10H17), 17H117-9 (*S. stoloniferum* PI 249929 × 10H17), and 17H131 (*S. stoloniferum* PI 498027 × 10H17); a BC_1_ plant, 18H255 [(*S. stoloniferum* PI 558450 × 10H17) × 10H17]; and a tetraploid cultivar, Alwara. Among them, 15H156, 18H225 and cv. Alwara are T-CMS clones, while 17H117-9 and 17H131 are pollen-fertile clones [33]. All the plants were grown in a greenhouse. No specific permissions or licenses were required for our collections and experiments. All methods were done in accordance with national, and international guidelines for plant experiments.

Total DNA was extracted from 1 g of young leaves using a Nucleobond HMW DNA kit (Macherey–Nagel, Germany), with minor modifications: the lysing time was extended to 3 h at 50 ℃. The extracted DNA was subjected to size selection using a Short Read Eliminator Kit (Circulomics, USA) and resuspended in 10 mM Tris–HCl buffer (pH 8.0) for seven days at 4 ℃. The quality of the DNA was assessed using a microspectrophotometer (NABI, MicroDigital, Korea). To detect mitochondrial DNA polymorphisms and perform sequencing analysis, DNA samples from 38 accessions of *S. stoloniferum* and 39 interspecific hybrids from *S. stoloniferum* × *S. tuberosum*, which were previously described in the study by Sanetomo and Nashiki [33], were used. For a broad survey of mitochondrial DNA polymorphisms, DNA samples from 129 accessions of 27 *Solanum* species described by Sanetomo and Hosaka [16] were used. For crossing with *S. verrucosum*, eight breeding clones, mostly those derived from *S. phureja* (15H138-1, 15H140-2, 97H32-6, 98H20-5, *S. phureja* 1.22, W6522195-19, W872209-36, and W872209-51), were used. Seeds of all accessions with a plant introduction (PI) number were obtained from the US Potato Genebank at Sturgeon Bay, Wisconsin, USA, with appropriate permissions.

### Library preparation and nanopore sequencing

For library preparation, high-molecular DNA was prepared for sequencing following the standard protocol of a Ligation Sequencing Kit (SQK-LSK109, Oxford Nanopore, UK). The reagent mixture consisting of 2 μl of NEBNext FFPE Repair Mix, 3.5 μl of NEBNext FFPE Repair Buffer, 3.5 μl of Ultra II End-prep Reaction Buffer, 3 μl of Ultra II End-Prep Enzyme Mix (NEBNext Companion Module for Oxford Nanopore Technologies Ligation Sequencing, NEB, UK), and 2 μg of DNA in 48 μl of 10 mM Tris–HCl buffer (pH 8.0) was incubated at 20 ℃ for 30 min and then at 65 ℃ for 30 min in a thermal cycler (GeneAMP ABI9700, Thermo Fisher Scientific, USA). The DNA was cleaned with the same volume of AMPure XP Beads (Beckman Coulter, USA), and the bead pellet was washed twice in 200 μl of 70% (w/w) ethanol in nuclease-free water. The supernatant was removed completely, and the pellet was subsequently dried at room temperature for 30 s. The bead pellet was resuspended in 61 μl of nuclease-free water. One microliter of eluted DNA was quantified using a QuantiFluor system (Promega, USA). For adapter ligation, 25 μl of LNB, 5 μl of AMX, 10 μl of NEBNext Quick T4 Ligase, and 60 μl of DNA were mixed together and incubated at 25 ℃ for 1 h. The ligated DNA was purified with 40 μl of AMPure XP Beads, and the bead pellet was washed twice with 250 μl of LFB by resuspension and pelleting on a magnet. After drying at room temperature for 30 s, the bead pellet was resuspended in 15 μl of EB buffer, and 1 μl of DNA sample was quantified using a QuantiFluor system. Finally, 37.5 μl of SQB, 25.5 μl of loading beads and the DNA library contents were mixed and loaded into a MinION flow cell R9.4 (FLO-MIN106SP and FLO-MIN106D). All sequencing processes were controlled with MinKNOW and run for 24 h, with the default parameters. Individual samples with low output were sequenced several times until enough outputs were obtained. The output data were saved in Fast5 format.

### Base calling, read preprocessing and de novo assembly

Fast5 files were base-called with Guppy ver. 3.3.3 (GPU and high-accuracy modes) on Ubuntu 16.04 LTS. Adapters were trimmed with Porechop (ver. 0.2.1, https://github.com/rrwick/Porechop). Low-quality reads (mean QS < 7) and short reads (length < 1500 bp) were removed using NanoFilt [81], and the preprocessed reads were analyzed by the same tool. To extract reads of the mitogenome, all the reads were mapped to the reference mitogenome sequences of cv. Désirée (MN104801, MN104802, and MN104803 [9]) with Minimap2 (with the -x map-ont option), and the mapped reads were extracted in BAM format using SAMtools ver. 1.10 [82]. To reduce computation time, the extracted reads were randomly sampled until enough coverage (50 × to 100 ×) was obtained using SeqKit ver. 0.11.0 [83]. The sampled reads were inputted for de novo assembly using Flye ver. 2.7 with the meta option [84], and contig sequences that did not show homology with the reference mitogenome sequences according to dot plot analysis using D-GENIES [85] were manually removed. Some collapsed contigs were joined manually using Bandage [86]. Assembled outputs were called for consensus three times with Racon ver. 1.4.3 [87] and polished with Pilon ver. 1.23 [88]; consensus calling and polishing were performed only for long reads. All computing was performed on a computer with Ubuntu 16.04 LTS as the operating system, an Intel Core i5 8400 CPU, 64 GB of RAM (DDR4-2400) and a 1 TB of SSD hard drive.

### Annotation and homology searches

Annotation of the organelle genomes was performed based on GeSeq [89]. For reference gene information, the dataset of three mitochondrial DNA molecules from cv. Désirée [9] was used. The selected parameters were “Linear model” and “Plastid (land plants)” for the sequenced source and “keep best annotation only” for the annotation revisions. Protein-coding DNA sequences (CDSs) and rRNA (and not tRNA) were annotated with > 25% protein search identity and > 50% rRNA and DNA search identity, respectively. A homology search was performed by BLAST analysis provided by the National Center for Biotechnology Information (NCBI).

### PCR amplification for the detection of mitochondrial polymorphisms and for sequencing

PCR primer sets were designed to amplify several inter- and intragenic mitochondrial genes: *cox2*-partial gene, *nad6*, *rpl5-ψrps14*, *rps1*, *rps4*, and *rps10* (Table S2). PCR amplification was carried out in a 10 μl reaction solution that included 2 μl of DNA (5 ng/μl), 5 μl of 2 × Ampdirect® Plus (Shimadzu, Japan), 0.25 U of heat-activated *Taq* DNA polymerase (LA Taq® Hot Start Polymerase, Takara Bio, Inc., Kusatsu, Japan), and forward and reverse primers (each at 0.3 μM). The thermal cycling profile was as follows: 95 °C for 10 min, followed by an initial cycle of 94 ℃ for 30 s, 65 ℃ for 30 s, and 72 ℃ for 1 min; a reduction in the annealing temperature by 1 ℃ during each of the next five cycles, followed by an annealing temperature of 60 ℃ for the remaining 25 cycles; and a final extension at 72 ℃ for 5 min. The amplification products were electrophoresed on a 2% agarose gel with 1 × TAE buffer (40 mM Tris, 20 mM acetic acid, and 1 mM EDTA). The PCR products were subjected to Sanger-sequencing by a commercial company (Takara Bio, Inc., Kusatsu, Japan).

### Real-time PCR for the detection of copy number variation

To detect copy number variation of mitochondrial DNA molecules, real-time PCR was performed using a LightCycler 96 System (Roche, Switzerland). A ten microliter solution that included 3 μl of DNA (5 ng/μl), 5 μl of FastStart Essential DNA Green Master Mix (Roche, Switzerland), and forward and reverse primers (each at 0.25 μM) was used (Table S2). The thermal cycling profile was as follows: 95 °C for 10 min; 45 cycles of 95 ℃ for 10 s, 60 ℃ for 10 s, and 72 ℃ for 10 s; and then 97 ℃ for 1 s for melting. The relative quantities were measured against that of the adenine phosphoribosyl transferase gene (*aprt*), a housekeeping gene encoded in the nuclear genome [41]. For each sample, the relative quantities of three replicates (with two tubes per reaction) were obtained and averaged.

### Transcriptome analysis

Total RNA from the pollen of *S. stoloniferum* PI 498035 and PI 558450 was extracted using an RNeasy® Plant Mini Kit (QIAGEN, Germany). The RNA was treated with DNase (TURBO DNA-*free*™, Ambion, TX, USA) for 20 min at 37 °C. Transcriptome analysis was then performed by a commercial service provider (Rhelixa, Japan). A library was prepared using a NEBNext® Poly(A) mRNA Magnetic Isolation Module (for PolyA selection) and a NEBNext Directional Ultra RNA Library Prep Kit for Illumina® (for strand-specific libraries). A 150 bp paired-end run was performed on an Illumina NovaSeq 6000 sequence platform. The reads were qualified using FastQC 0.11.8 [90] and MultiQC v1.8 [91] and then filtered using Trimmomatic 0.38 [92] with “ILLUMINACLIP:TruSeq3-PE.fa:2:30:10 LEADING:20 TRAILING:20 SLIDINGWINDOW:4:15 MINLEN:36” options. The trimmed reads were mapped to the mitochondrial genome sequences of cv. Alwara using HISAT2 ver. 2.1.0 [93]. The reads mapped to the intergenic region between *rpl5-ψrps14* and *nad6* were subsequently extracted by SAMtools ver. 1.9 [82] and visualized by IGV [94].

## Supplementary Information

**Additional file 1: Table S1.** Summary statistics of MinION sequencing. ** Table S2.** PCR markers. **Table S3.** Characterization of interspecific hybrids generated from crosses between *S. stoloniferum* and *S. tuberosum* accessions. **Table S4.** Pollen transcript reads mapped to the region between *rpl5*-*ψrps14* and *nad6*. **Table S5.** Survey of P-3 and P-4 regions in a wide range of tuber-bearing *Solanum* species. **Table S6.** Mitogenome sequence data are available under BioProject No. PRJDB12191.

**Additional file 2: Figure S1.** Dot-plot alignments of assembled contigs vs. the reference mitogenome (MN104801 and MN104802) of cv. Désirée. **Figure S2.** Sequence of the intergenic region between *rpl5*-*ψrps14* and *nad6* (P-3) in cv. Alwara. **Figure S3.** Sequence of the intergenic region between *rpl1* and *cox2*-*partial* (P-5) in cv. Alwara. **Figure S4.** Photographs of full-length electrophoresed gels used for Figure 3. Stoichiometric differences between P-3 and the 859-bp band via **a** rpl5rps14outF/nad6 and **b** rpl5rps14outF/ALM5 (for the 859-bp band) primer sets.

**Additional file 3.** Gene annotation data. 

## Data Availability

All the data generated or analyzed during this study are included in this published article and its supplementary information files. Wild species accessions with a plant introduction (PI) number are available from the US Potato Genebank at Sturgeon Bay, Wisconsin. The other clones and varieties are available from the corresponding author. The genome sequence data that support the findings of this study are available in GenBank of DDBJ at https://www.ddbj.nig.ac.jp/ddbj/index-e.html under accession numbers LC649808–649830. The associated BioProject number is PRJDB12191, and the BioSample numbers are SAMD00400419–00400421 and SAMD00402078–00402079 (Table S6). RNA sequence data is available in PRJDB13350, and the BioSample numbers are SAMD00452379–SAMD00452382.

## Abbreviations

CMS: Cytoplasmic male sterility
Mitogenome: Mitochondrial genomes
T-CMS: Tetrad sterility-type cytoplasmic male sterility
Orf: Open Reading Frame
PCR: Polymerase chain reaction
PMC: Pollen mother cell
RC: Recombinant contig
