# Supplementary material for: Discovery of a novel mitochondrial DNA molecule associated with tetrad pollen sterility in potato
Source: BMC Plant Biol. 2022 Jun 21;22:302. doi: 10.1186/s12870-022-03669-8 (PMC9210639; doi:10.1186/s12870-022-03669-8)
Supplement: Supplementary file 2 — Additional file 2: Figure S1. Dot-plot alignments of assembled contigs vs. the reference mitogenome (MN104801 and MN104802) of cv. Désirée. Figure S2. Sequence of the intergenic region between rpl5-ψrps14 and nad6 (P-3) in cv. Alwara. Figure S3. Sequence of the intergenic region between rpl1 and cox2-partial (P-5) in cv. Alwara. Figure S4. Photographs of full-length electrophoresed gels used for Figure 3. Stoichiometric differences between P-3 and the 859-bp band via a rpl5rps14outF/nad6 and b rpl5rps14outF/ALM5 (for the 859-bp band) primer sets. [file 12870_2022_3669_MOESM2_ESM.pptx]

## Slide 1
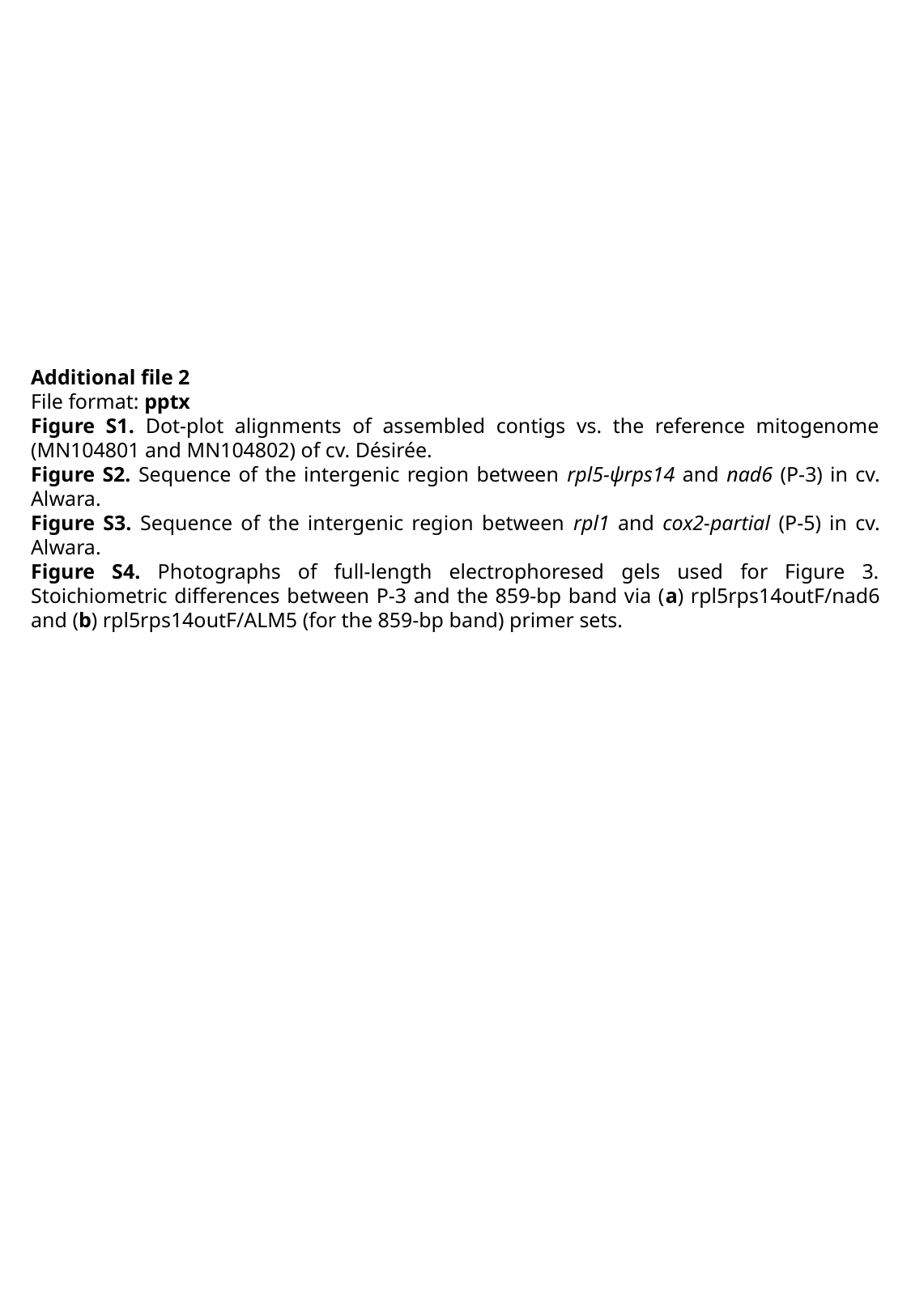

Additional file 2
File format: pptx
Figure S1. Dot-plot alignments of assembled contigs vs. the reference mitogenome (MN104801 and MN104802) of cv. Désirée.
Figure S2. Sequence of the intergenic region between rpl5-ψrps14 and nad6 (P-3) in cv. Alwara.
Figure S3. Sequence of the intergenic region between rpl1 and cox2-partial (P-5) in cv. Alwara.
Figure S4. Photographs of full-length electrophoresed gels used for Figure 3. Stoichiometric differences between P-3 and the 859-bp band via (a) rpl5rps14outF/nad6 and (b) rpl5rps14outF/ALM5 (for the 859-bp band) primer sets.

## Slide 2
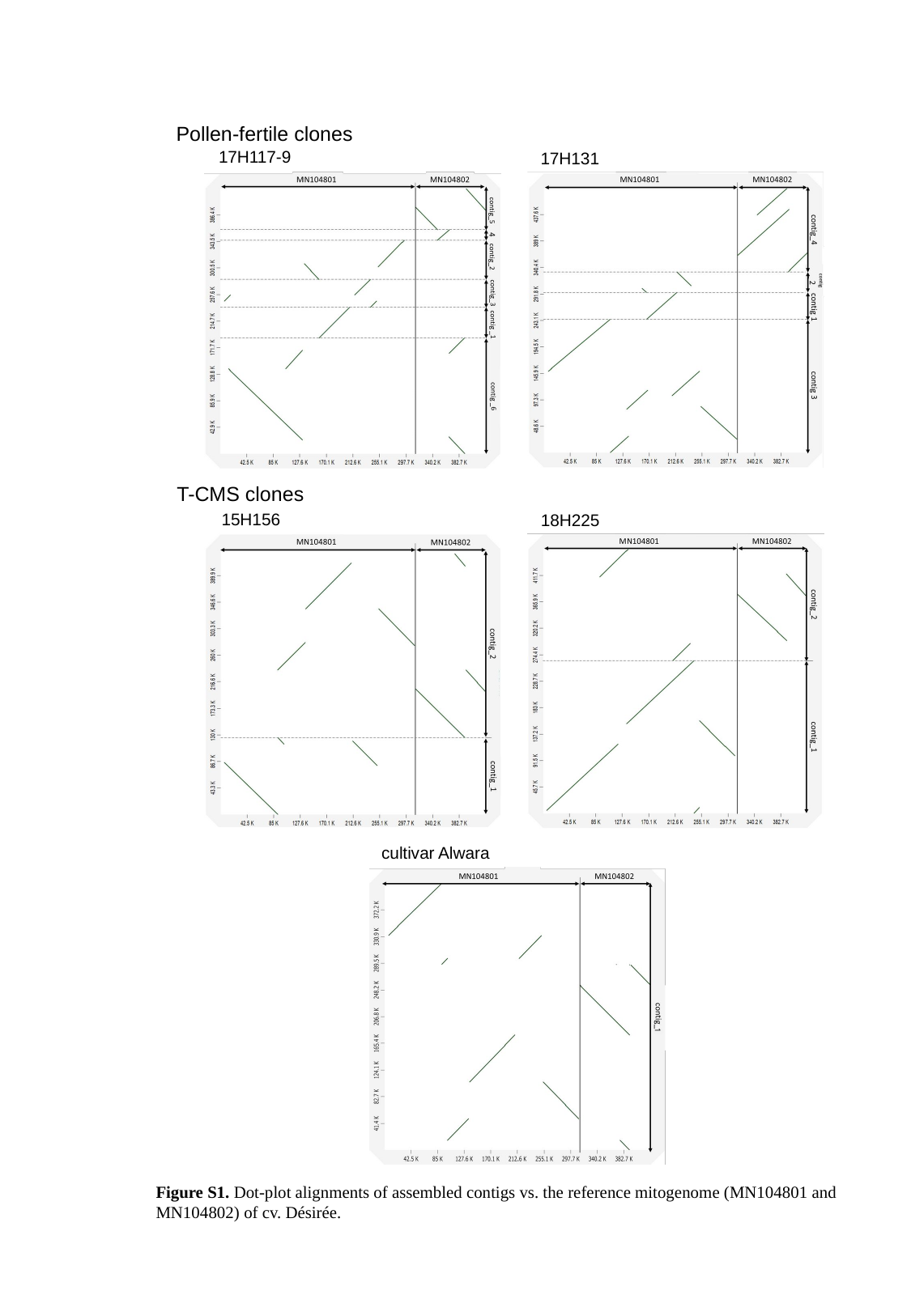

Pollen-fertile clones
17H117-9
17H131
T-CMS clones
15H156
18H225
cultivar Alwara
1
Figure S1. Dot-plot alignments of assembled contigs vs. the reference mitogenome (MN104801 and MN104802) of cv. Désirée.

## Slide 3
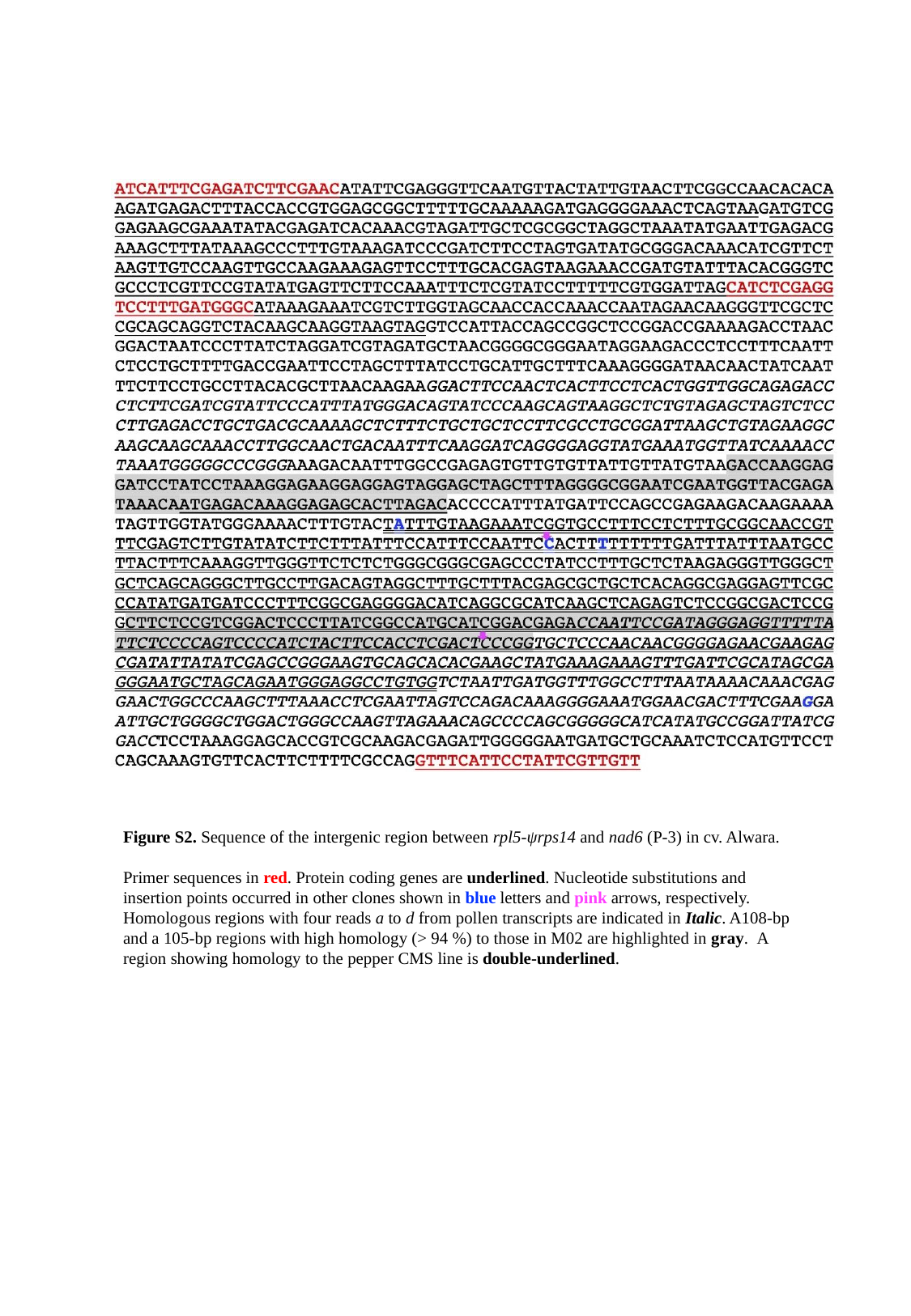

Figure S2. Sequence of the intergenic region between rpl5-ψrps14 and nad6 (P-3) in cv. Alwara.
Primer sequences in red. Protein coding genes are underlined. Nucleotide substitutions and insertion points occurred in other clones shown in blue letters and pink arrows, respectively. Homologous regions with four reads a to d from pollen transcripts are indicated in Italic. A108-bp and a 105-bp regions with high homology (> 94 %) to those in M02 are highlighted in gray. A region showing homology to the pepper CMS line is double-underlined.

## Slide 4
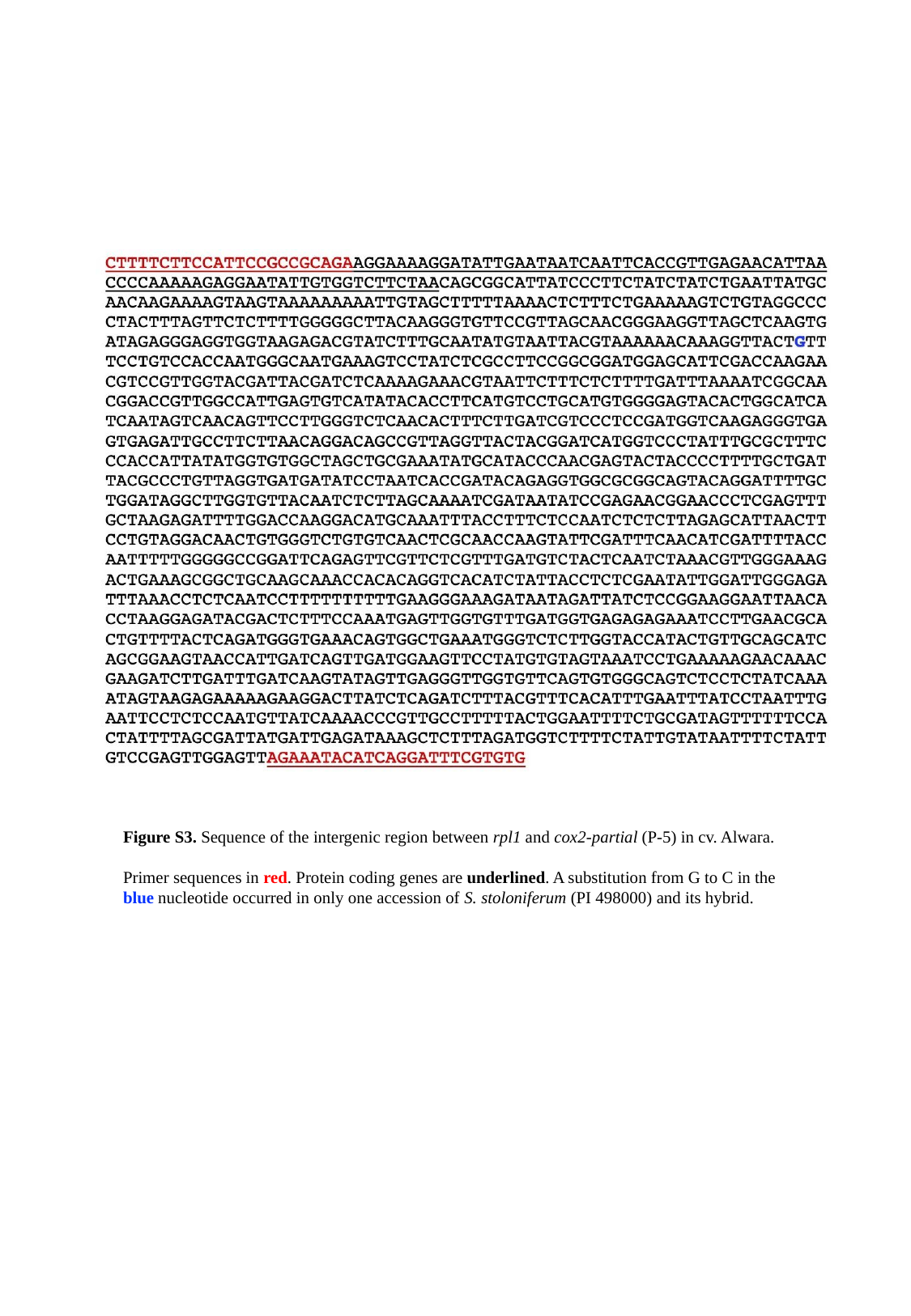

Figure S3. Sequence of the intergenic region between rpl1 and cox2-partial (P-5) in cv. Alwara.
Primer sequences in red. Protein coding genes are underlined. A substitution from G to C in the blue nucleotide occurred in only one accession of S. stoloniferum (PI 498000) and its hybrid.

## Slide 5
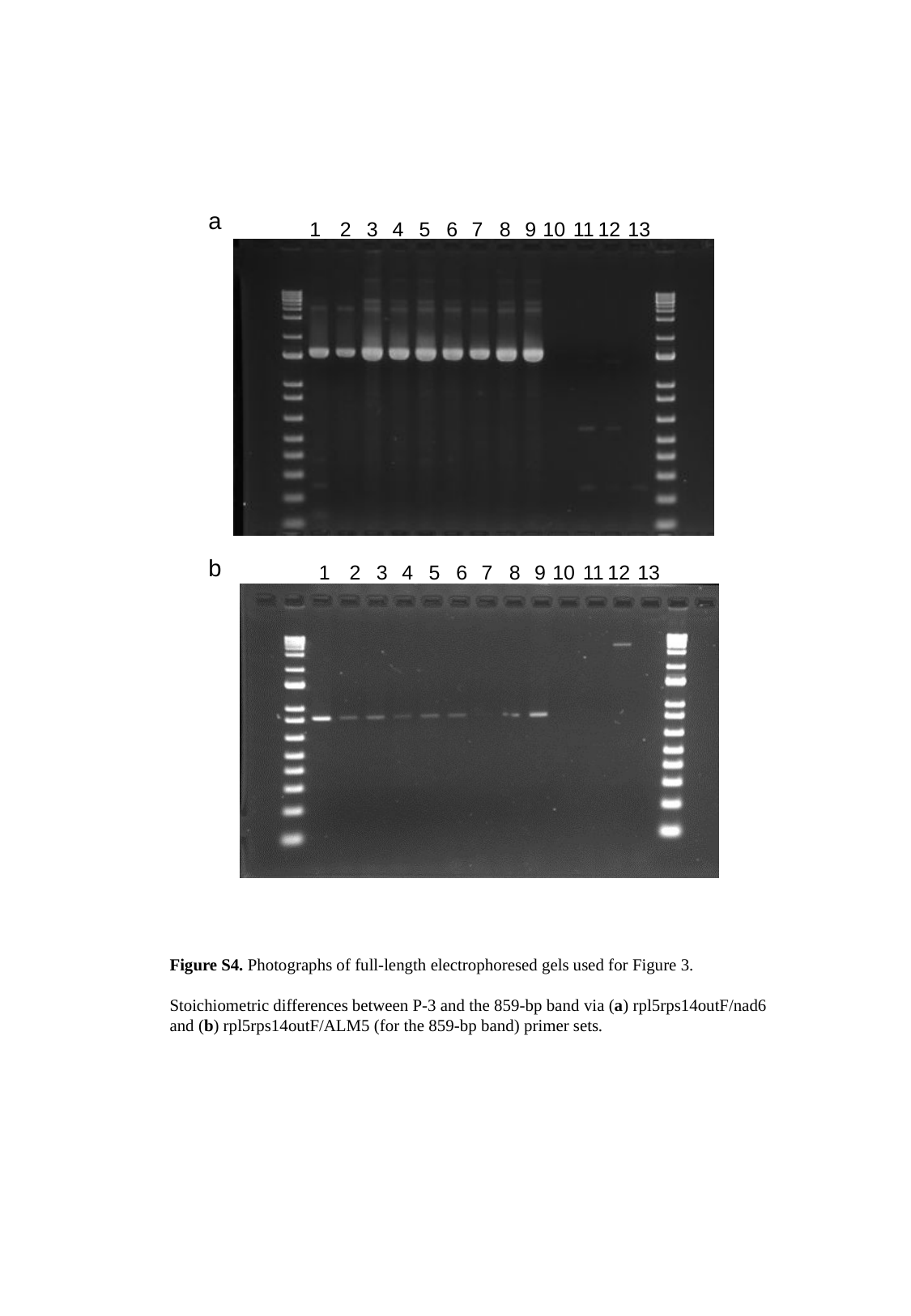

a
1
2
3
4
5
6
7
8
9
10
11
12
13
b
1
2
3
4
5
6
7
8
9
10
11
12
13
Figure S4. Photographs of full-length electrophoresed gels used for Figure 3.
Stoichiometric differences between P-3 and the 859-bp band via (a) rpl5rps14outF/nad6 and (b) rpl5rps14outF/ALM5 (for the 859-bp band) primer sets.
